# Harnessing of Sunflower Stalks by Hydrolysis and Fermentation with *Hansenula polymorpha* to Produce Biofuels

**DOI:** 10.3390/polym16243548

**Published:** 2024-12-19

**Authors:** Mª Lourdes Martínez-Cartas, Manuel Cuevas-Aranda, Sebastián Sánchez

**Affiliations:** 1 Department of Chemical, Environmental and Materials Engineering, Higher Polytechnical School of University of Jaén, Avda. de la Universidad s/n, 23700 Linares, Spain; mcuevas@ujaen.es (M.C.-A.); ssanchez@ujaen.es (S.S.); 2 University Institute of Research in Olive Grove and Olive Oils, University of Jaén, Science and Technology Park GEOLIT, 23620 Mengíbar, Spain

**Keywords:** ethanol, fermentation, hydrolysis, *Hansenula polymorpha*, nitric acid, enzymes, xylitol

## Abstract

A sequential valorization process of sunflower stalks was carried out using nitric acid (0.1–2 mol dm^−3^) as a hydrolytic agent and fermenting the hydrolysate of higher sugar concentration in the presence of the non-conventional yeast *Hansenula polymorpha*. Values reached for ethanol yield (0.25 g g^−1^) and xylitol yield (0.14 g g^−1^) were higher than those achieved after pretreatment with other acids in previous studies. The effect of acid treatment with nitric, phosphoric, and sulfuric acids on the separated solid fractions was evaluated to determine its potential use as solid biofuel by FTIR and SEM determinations. A significant loss of lignin and hemicellulose was found in the solid treated with nitric acid, while a higher HHV was obtained when pretreated with phosphoric acid (19.16 MJ kg^−1^) and sulfuric acid (19.12 MJ kg^−1^). A subsequent enzymatic hydrolysis of acid-pretreated solids showed that the nitric acid pretreatment increased the availability of glucose from the cellulose fraction to a greater extent than the other two acids, by reducing the hemicellulose fraction to 0.7% and the lignin fraction to 2.5%. This study shows that pretreatment of biomass with nitric acid leads to better fermentation results to obtain biofuels such as ethanol, which could be further increased by additional enzymatic hydrolysis, while pretreatment with the other two acids generates better solid fuels.

## 1. Introduction

There is a deep interconnectedness between the energy and food systems. Agri-food systems consume about 30% of the world’s energy; moreover, energy is responsible for one-third of the greenhouse gas emissions from these systems. This highlights the need for revitalization of these agri-food systems by guaranteeing the availability of reliable, affordable, and environmentally sustainable energy for primary production with post-harvest processing as a key factor for reaching higher yields, higher revenues, lower losses, and greater climate improvements [1]. In the same vein, biomass demand could increase substantially after 2030, particularly in relation to advanced biofuels, to reach the 2050 target (−80% GHG) [2]. In this sense, the aim of a biofuel’s transport policy should be to create, as soon as possible, a more economic and sustainable alternative to oil [3]. In this context, the lignocellulose crop potential is estimated only on unused, abandoned, and degraded lands. The estimated amount of lignocellulosic crops’ potential for 2030 ranges from 36 to 108 million tons, and this value for 2050 ranges from 42 to 127 million tons [4]. Considering that Ukraine, EU-27, and the Russian Federation account for 56% of world exports of sunflower seeds [5] (and specifically, in 2020, 8.7 × 10^6^ tons sunflower stalks derived from this crop in Europe and 8.8 × 10^5^ tons in Spain [6]), these lignocellulose wastes constitute a relevant resource of potentially recoverable biomass.

Among the different uses of lignocellulose wastes [7], an efficient pathway to produce fermentable sugars is the acid hydrolysis treatment [8]. The highest degree of polymerization among lignocellulosic polymers is cellulose, which is insoluble in water, although the monomer (D-glucose) and short oligomers are water-soluble. Hemicellulose consists of hexose and pentose sugars. Lignin has been identified as one of the main hurdles to an energy-efficient biomass decomposition process [9]. The cell wall of the plant is made up of cellulose, hemicellulose, and lignin, forming a very compact structure that is stable and resistant. Therefore, considerable mechanical, chemical, or biological forces are required to separate these major components. Acid pretreatment is a highly effective chemical method used to break the lignocellulosic matrix by cleavage of glycosidic bonds [10]. An important advantage of using concentrated acid pretreatment (such as sulfuric acid, phosphoric acid, and nitric acid, among others) to achieve high yields of sugars is to avoid the use of enzymes by means of a saccharification stage. However, byproducts obtained from the acid pretreatment of the lignocellulose fraction could generate compounds with an inhibitory effect on the fermenting microorganism. Common aliphatic carboxylic acids, e.g., acetic acid, formic acid, and levulinic acid, as well as furfural and hydroxymethyl furfural (HMF), are carbohydrate breakdown products, which have relatively low toxicity but may be generated at high concentrations, depending on pretreatment conditions and the raw material [11]. The total or partial solubilization of the hemicellulose or cellulosic fractions by acid hydrolysis of the residue in question may result in sugar solutions. Pentose sugars D-xylose and L-arabinose are two of the most abundant sugars, occupying the second and third positions for abundance in lignocellulosic biomass. The metabolism and bioconversion of these pentoses have, therefore, been studied extensively. Several different pathways of D-xylose and L-arabinose catabolism in bacteria and yeasts are known [12].

The use of yeasts capable of fermenting both hexoses and pentoses, during a subsequent fermentation process of these hydrolysates, may give rise to byproducts of industrial interest such as ethanol, xylitol, and antioxidants. Different microorganisms like bacteria [13], fungus [14], and yeasts [15,16] can be used in the fermentation process to generate bioproducts. Among them, *Hansenula polymorpha* is a non-conventional yeast with interesting biotechnological applications [17]. Its specific properties (thermotolerance [18] inhibition, dehydration resistance [19,20] and high methanol toleration [21]) make it an ideal microorganism to be used in media with a high presence of inhibitors, such as those generated after acid hydrolysis of lignocellulosic biomass. Although sugars are used here as a carbon source, *H. polymorpha* is described as a methylotrophic yeast (which is able to use methanol as a source of carbon and energy) [22] that increases its biosynthetic potential to obtain valuable chemicals, namely, proteins, polyketides, fatty acids, and isoprenoids. Furthermore, *H. polymorpha* could be characterized by utilization of both D-glucose and D-xylose in the alcoholic fermentation [23].

It has been found that the structure of purified cellulose could be modified more than in the case of biomass during an acid pretreatment and that biomass may be a better substrate than original biopolymers to evaluate structural changes occurring during the industrial process. Similarly, the chemical modification of carbohydrates, depending on the availability of biologically degradable ones, or their accessibility, among other factors, could undermine the effectiveness of enzymatic hydrolysis [24]. In this sense, the combination of acid pretreatment with enzymatic hydrolysis could be an appropriate procedure to boost the availability of fermentable sugars. Similarly, it has been reported that glucose yields from enzymatic hydrolysis were strongly affected by acid pretreatment, with maximum glucose recovery (72.8%) being achieved with diluted sulfuric acid pretreatment at high temperatures [25]. Although a widespread methodology of lignocellulosic waste recovery could be thought of, it is not possible to develop a universal pretreatment method of equivalent efficiency for all types of biomass, taking into account the great variability in the composition and structure of these materials. Therefore, for each type of material, we must select a pretreatment method (or a combination of them) [26,27].

This work intended to emphasize the promising potential of sunflower stalk residues as a lignocellulosic resource to provide biofuels. For this, a sequential treatment was carried out. In this treatment, the first stage was the acid hydrolysis of sunflower stalks to obtain a liquid fraction (hydrolysate), which was fermented in a second stage to determine the possibility of obtaining ethanol, xylitol, and other byproducts such as phenolic compounds. The focus of this work is on nitric acid as a hydrolytic agent, given that the study of hydrolysis and fermentation with acids, such as phosphoric acid (H_3_PO_4_) [28] and sulfuric acid (H_2_SO_4_) [29], were executed in previous works, comparing the results obtained. In the case of the solid fraction, the study was performed using SEM and FTIR analysis to observe the effect that acid treatment (using three acids: nitric, sulfuric, and phosphoric acid) exerts on the raw material to obtain solid biofuels, which we will evaluate with the determination of HHVs (higher heating value).

A final enzyme hydrolysis stage was applied on the pretreated solids with the three acids, allowing us to study how to increase the generation of glucose contained in the cellulosic fraction of the residues pretreated with acid and, thus, increase the source of carbon for subsequent fermentations of these enzymatic hydrolysates. We, thus, present a procedure for the harnessing of sunflower stalk residues for the production of biofuels and byproducts, such as xylitol.

## 2. Materials and Methods

### 2.1. Raw Material and Acid Pretreatment

The sunflower stalk waste was compiled at a farm in the province of Jaen (Spain), and was ground with a cutting mill and screened using a sieve machine to obtain the necessary size, 0.425–0.6 mm. The raw material was characterized, measuring the content of moisture [30], mineral matter [31], acid detergent fiber (ADF), neutral detergent fiber (NDF) [32], and lignin [33]. The hemicellulose content was determined by the difference between %NDF and %ADF, as well as the difference between the percentage of FAD and lignin, allowing us to calculate the percentage of cellulose. The values obtained were 8.10% moisture, 16.60% hemicellulose, 50.30% cellulose, 13.40% lignin, and 4.34% ash. The acid pretreatment was performed in a stirred batch reactor of 1 dm^3^ capacity, with 50 g of sunflower stalk, a liquid/solid relationship of 20/1 (1000 mL of acid/50 g solid), and a temperature of 99 °C. The hydrolysis time was set to 240 min and agitation speed of 200 rpm. A Teflon (PTFE) stirrer with anchor-type ending was used. Concentrations of nitric acid (C_Ao_) were varied within the interval 0.0–2.0 mol dm^−3^. During the pretreatment, samples of 5 mL were taken and analyzed in order to determine the variation in sugar concentrations. At the end of the acid prehydrolysis, the reactor contents were filtered under vacuum to separate the liquid prehydrolysate and the pretreated solids, which were washed with water to neutral pH. The solids were dried at room temperature and they are referred to as “acid pretreated solids” (APSs), while the liquid prehydrolysates were subjected to a conditioning operation. On the other hand, acid prehydrolysis was also realized under the same conditions mentioned, but using sulfuric acid (0.5 mol dm^−3^) and phosphoric acid (2.6 mol dm^−3^) [28,29]. These concentrations were set, according to previous publications, to obtain the highest yields of reducing sugars in the prehydrolysates.

### 2.2. Prehydrolysate Characterization

The acid prehydrolysates obtained were analyzed by the determination of total reducing sugars (TRS) [34] and D-glucose [35]. Concentrations of L-arabinose, D-galactose, D-glucose, and D-xylose were determined by high-performance ionic liquid chromatography (HPILC) employing an ionic chromatograph (Dionex ICS-4000, Puerto Rico, USA) with a pulsed amperometric detector with a gold electrode [29]. The determination of phenolic compounds concentration was made by high-performance liquid chromatography (HPLC) using an LC/MS chromatograph (Agilent 6400, Lexington, MA, USA) with a mass spectrometer detector and triple quadrupole.

### 2.3. Prehydrolysates Conditioning

For all the prehydrolysates obtained, the pH was regulated using concentrated KOH (10 mol dm^−3^) until a pH of 4.5 was reached. Subsequently, the prehydrolysates were concentrated using a Büchi rotary evaporator (Büchi mod. R114, Barcelona, Spain). Although the temperature did not exceed 40 °C, the increased concentration and heating in the rotary evaporator led to a darkening of the hydrolysate. To minimize this intense color, a bleaching stage was applied, adding to the solution an activated carbon in pellets (number 2), in an amount corresponding to 8% by weight. The mixture was stirred at 35 °C for 1 h.

Lastly, to separate the activated carbon, the liquid medium was filtered first through paper filter and after with a peristaltic pump to push the liquid forward through a cellulose nitrate membrane (porous size of 0.45 µm) using polycarbonate filters.

### 2.4. Fermentation Process Description

The fermentation process was carried out with the yeast provided by American Type Culture Collection, *Ogataea* (*Hansenula*) *polymorpha* ATCC 34438, whose taxonomy indicates that *O. thermophila* is a synonym of *H. polymorpha* or *Pichia angusta* [36], one of the most relevant non-conventional yeasts with industrial applications [37]. The microorganism was manipulated and transferred as inoculum along with the medium Lindegren [38] to the fermentation installation, as was described in previous work [28].

The reactor was inoculated with 0.1 g dm^−3^ of yeast, fixing the temperature to 30 °C, the pH to 5.5, and the agitation to 500 rpm. During the fermentation process, samples of 5 cm^3^ were taken and analyzed to determine the variation in pH and the biomass and sugars concentrations during the culture, just as the xylitol [39], ethanol [40], and acetic acid [41] concentrations generated. The yeast biomass concentration was measured using a spectrophotometer (Biochrom Libra S60, Cambridge, UK). The standard calibration curve, relating the biomass concentration to the wavelength at 620 nm, was determined previously. All analyses were conducted in duplicate.

### 2.5. Solid Fraction Characterization

The pretreated solids were analyzed for the following parameters: moisture [30], mineral matter [31], and the hemicellulose and cellulose content, which were calculated in the manner previously described in the characterization of the raw material. In addition, the following analytical techniques were applied.

#### 2.5.1. Fourier Transform Infrared (FTIR) Equipment

FTIR spectra were determined using a Vertex 70 spectrophotometer (Bruker, Billerica, MA, USA). A KBr-disc method in the range of 4000–400 cm^−1^ with a resolution of 4 cm^−1^ and 100 scans was used.

#### 2.5.2. Elemental Analysis

To determine the carbon, hydrogen, nitrogen, and sulfur content according to Standard [42], a CHNS elemental analyzer (Thermo Finnigan Flash EA1112, Waltham, MA, USA) was used. The oxygen content was calculated by subtracting the CHN content from the total. The determinations were performed in duplicate.

#### 2.5.3. Heating Values

The experimental determination of the higher heating values (HHVs) of the raw material and pretreated samples were conducted according to Standard [43]. To this end, an automatic isoperibol calorimeter (Parr series 6400, Moline, IL, USA) was used.

#### 2.5.4. SEM Observation

The microstructures of all the samples were examined using a high-resolution scanning electronic microscope (FE-SEM) Merlin (Zeiss, Oberkochen, Germany). For this observation, all samples were first covered with carbon.

### 2.6. Enzymatic Hydrolysis Description

An enzymatic hydrolysis treatment was applied to the raw material and solid fractions separated after acid pretreatments (APSs) using a cellulolytic complex (Celluclast 1.5 L) provided by Novo Nordisk Bioindustrial (Madrid, Spain) with an enzymatic activity of 33.04 FPU cm^−3^ [25]. The enzyme loads tested were 10 FPUg^−1^ APS and 20 FPU g^−1^ APS. The dose of β-glucosidase was 30 international units (IUs) per gram of dry APS. To avert microbial growth and consumption of the released sugars, an antibiotic (chloramphenicol) was mixed at a final concentration of 50 μg cm^−3^. The enzyme treatment conditions were 50 °C on a rotary shaker at 150 rpm for 96 h and at a solid material concentration of 10% (w/v), regulating the pH to 4.8 with sodium citrate buffer 0.05 mol dm^−3^. Samples were extracted from the reaction media at different times (24, 48, 72, and 120 h) to determine the D-glucose and xylose concentration using the HPILC previously described (in Section 2.2). In the case of raw material without enzyme (RMEW), only the experiment to 120 h was measured. From these values, enzyme hydrolysis yields were calculated as grams of glucose by enzymatic hydrolysis per 100 g glucose in the substrate. After completion of the enzymatic hydrolysis, the content of each reactor was filtered under vacuum and washed with water to determine, gravimetrically, the percentage of solid recovered. All essays were conducted in duplicate.

## 3. Results and Discussion

### 3.1. Influence of Nitric Acid Concentration on Prehydrolysate Composition

**Figure 1:**
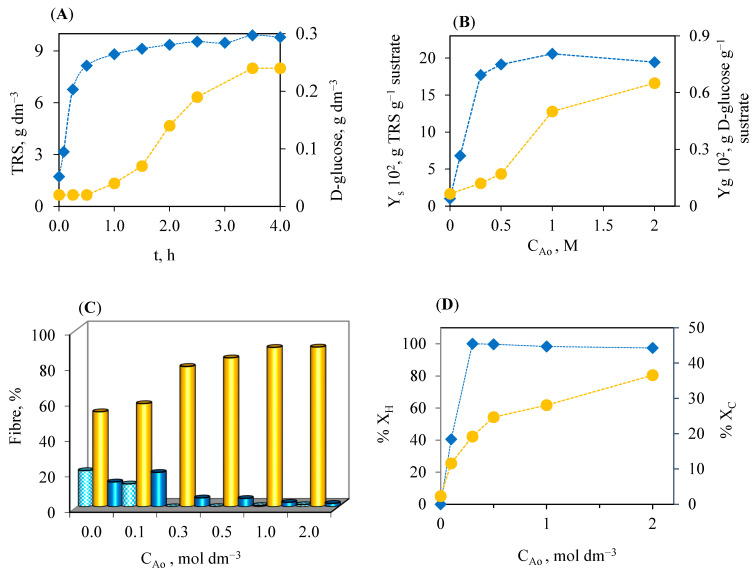
(**A**) Variation in the concentrations of TRS (♦) and D-glucose (●) produced throughout hydrolysis process with nitric acid 1.0 M. (**B**) Total reducing sugars (TRSs) (♦) and D-glucose (●) yields (referred to initial dry material) versus initial concentration of nitric acid used in the hydrolysis process. (**C**) Variation in the hemicellulose (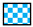), cellulose (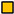) and lignin (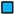) percentages with the initial concentration of and nitric acid used in the hydrolysis process. (**D**) Influence of nitric acid concentrations on hemicellulose (♦) and cellulose (●) fractional conversion.

Acid prehydrolysis of sunflower stalks was performed with nitric acid of concentration in the 0–2 mol dm^−3^ range to determine the amount of catalyst for which the maximum production of fermentable sugars is achieved. Prehydrolysis was made for 4 h to ensure adequate contact between the biomass and the acid. Figure 1A shows the evolution of the concentration of TRS and glucose in the test performed with 1.0 mol dm^−3^ nitric acid, reaching maximum concentrations of 9.8 g dm^−3^ (TRS) and 0.24 g dm^−3^ (glucose). Considering TRS yields, expressed as g TRS g^−1^ substrate, the larger yield value (0.2 g sugars g^−1^ substrate, equivalent to a concentration of 10 g TRS dm^−3^) was reached with a catalyst concentration of 1 mol dm^−3^. This yield value decreased slightly at the 2 mol dm^−3^ concentration (Figure 1B). Comparing these results with those obtained from the same raw material but using sulfuric and phosphoric acids as catalysts [28,29], the better result (0.29 g TRS g^−1^ substrate: 14.5 g TRS dm^−3^) was reached with the hydrolysate of H_2_SO_4_ 2.5 mol dm^−3^ [29] (Figure S1a and Figure S2a). In the case of H_3_PO_4_ [28], concentrations higher than 2 mol dm^−3^ were necessary to obtain higher sugar yields than those obtained with nitric acid (Figure S2a). The longer sugar yields to lower concentrations of acid were achieved in the case of HNO_3_ 1 mol dm^−3^, 9 g TRS dm^−3^ (Figure 1B).

The D-glucose yields obtained in the case of nitric acid 1 mol dm^−3^ were slightly higher than those obtained with phosphoric of a bigger concentration (2.7 mol dm^−3^), reaching 0.2 g dm^−3^ of D-glucose after time of hydrolysis (Figure S2b). When working with sulfuric acid, the yields were increased to a greater extent from a concentration of 0.5 mol dm^−3^ sulfuric acid compared to the other two acids. It should be noted that with 2.5 mol dm^−3^ (13.3%) H_2_SO_4_, the highest value of D-glucose obtained was 1.1 g dm^−3^, after 4 h of hydrolysis (Figure S2b). 

Some authors, found that the sugars obtained from corncob hydrolysis in dilute phosphoric acid, generated under mild hydrolysis conditions, were mainly trehalose, L-arabinose, D-glucose, and D-fructose from hemicellulose and cellulose degradation. While, wild D-xylose was obtained under the most h hydrolysis conditions [44].

In relation to D-glucose concentration, when using nitric acid as a catalyst, the smallest concentrations of D-glucose were obtained for up to 3 h of hydrolysis, in which the results were equated with those obtained with the other two acids, around 0.25–0.30 g dm^−3^ (Figure S1b and Figure 1A). The hemicellulose fraction disappeared completely at the concentration of 0.3 mol dm^−3^ nitric acid, achieving the higher cellulosic fraction at the 1 mol dm^−3^ concentration (Figure 1C). This fact does not occur in the case of phosphoric acid, remaining a 4.2% of the hemicellulose even at the more elevated concentration of 2.67 mol dm^−3^ [28] (Figure S3). When working with another raw material, after 1 h of treatment with 0.6% nitric acid (approximately 0.24 mol dm^−3^) at 150 °C, over corn stover, a prehydrolysate with 22.01 g dm^−3^ xylose, 1.91 g dm^−3^ glucose, 2.90 g dm^−3^ arabinose, 2.42 g dm^−3^ acetic acid, and 0.21 g dm^−3^ furfural was obtained [45]. In this case, they needed to increase up to 150 °C to double the TRS content.

A determination of the fractional conversion, referring to the hemicellulose, cellulose, and lignin fractions, to hydrolysis processes with nitric acid was conducted, noting that the fractional conversion of cellulose is wider for nitric acid hydrolysis at inferior concentrations (less than 1 mol dm^−3^) (Figure 1D), when this value is compared with the values reached with phosphoric [28] and sulfuric acid [29] hydrolysis (Figure S4b). For higher concentrations of sulfuric acid, it is possible to achieve fractional conversions in cellulose close to 75%. A total conversion of the hemicellulose to the sulfuric acid concentration of 0.5 mol dm^−3^ was obtained, making it necessary to reach the acid concentration of 2.5 mol dm^−3^ to obtain the maximum value in the conversion of the cellulose fraction (Figure S4).

#### 3.1.1. Inhibitors

In relation to the fermentation inhibitors present in the detoxified acid prehydrolysate, the concentrations of different phenolic compounds and acetic acid are shown in Table S1. Of note is the low total concentration of polyphenols in the liquid medium (0.03 mg dm^−3^), as well as the moderate presence of acetic acid (2.1 g dm^−3^). Hydroxytyrosol (0.013 mg dm^−3^) and syringic acid (0.009 mg dm^−3^) were the most prominent polyphenols. These values are significantly lower than those obtained using the same raw material, by applying acid hydrolysis with other acids [28,29], which could facilitate the fermentation process of the sugars obtained with nitric acid. Considering that phenolic compounds formed from lignin could be breaking down membranes, they could tamper with intracellular hydrophobic function [46].

#### 3.1.2. Kinetics of the Acid Hydrolysis

**Figure 2:**
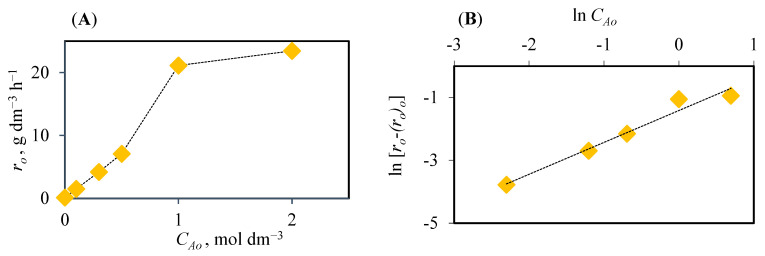
(**A**) Variation in the initial hydrolysis rates with the nitric acid concentrations. (**B**) Fit of the values of initial hydrolysis rate and initial nitric acid concentration to linearization of Equation (3).

The hydrolysis rate (r) during the pretreatment with nitric acid was determined from the values of the concentration of total reducing sugars, following the same kinetics model described in previous works [28,29]. Using the Equation (1) and its subsequent linearization (Equation (2)), the determination of the initial hydrolysis rate (r_o_) was carried out.

(1) $$s-s_{o}=\frac{S_{m}\;t}{\frac{S_{m}}{r_{o}}+t}$$

(2) $$\frac{1}{S-S_{o}}=\frac{1}{S_{m}}+\frac{1}{r_{o}}\frac{1}{t}$$

where *s_o_* = initial TRS concentration (g dm^−3^), *s_m_* = maximum TRS concentration (g dm^−3^), *r_o_* = initial hydrolysis rate (g dm^−3^ h^−1^), and *t* = time (h). With the method of initial rates, the values of the kinetic constant, *k*, and the apparent reaction order, n, were determined after the linearization of Equation (3).

(3) $$\left[r_{o}-({r_{o})}_{o}\right]=kC_{A_{o}}^{n}$$

where (*r_o_*)*_o_* represents the initial rate of hydrolysis without acid. Figure 2A shows the initial reaction rate value as a function of the nitric acid concentration, noting that the highest value of *r_o_* (23.46 g dm^−3^ h^−1^) was obtained for the maximum catalyst concentration (2 mol dm^−3^). Using the same raw material, its pretreatment with phosphoric acid led to a value of (*r_o_*)*_max_* of 3.5 g TRS dm^−3^ h^−1^ for a catalyst concentration of 1.67 mol dm^−3^ [28], while with sulfuric acid, the maximum value of (*r_o_*)*_max_* (2. 76 g TRS dm^−3^ h^−1^) was reached at a concentration of 0.5 mol dm^−3^ [29]. In addition, the linearization of Equation (3) allowed the determination of the apparent reaction order (*n* = 1.01) and the kinetic constant (*k* = 14.64 h^−1^).

### 3.2. Fermentation

The fermentation process was accomplished using *Ogataea* (*Hansenula*) *polymorpha*, ATCC 34438, and the hydrolysate obtained with nitric acid 1 mol dm^−3^ (corresponding to maximum TRS concentration), and submitted to detoxification.

#### 3.2.1. Biomass Production

Biomass production was evaluated by calculating the maximum specific growth rate (*μ_m_*) and biomass volumetric productivity (*P_b_*). During a fermentation process, the exponential growth phase can be quantified by the maximum specific growth rate (Equation (4)). In the case of fermentation with nitric acid, the *μ_m_* value was 0.12 h^−1^, a substantially superior rate to that achieved with phosphoric and sulfuric acids (0.008 h^−1^ and 0.022 h^−1^, respectively) [28,29].

(4) $$\ln(\frac{x}{x_{o}})=\mu_{m}t+a$$

Some authors have found that the chronological lifespan of *H. polymorpha* is strongly enhanced when cells are grown on methanol or ethanol, and the short lifespan of D-glucose is primarily caused by the medium acidification [47,48]. Using a synthetic medium and with *H. polymorpha* at 30 °C, other authors [49] obtained a value of *μ_m_* = 0.26 h^−1^. When they used hydrolysate without conditioning, the maximum specific growth rate was 0.062 h^−1^, less than that reached in the nitric acid hydrolysate. When working in the presence of the same yeast in synthetic medium at 40 °C, bigger values of *μ_m_* (0.175 h^−1^) were found [50].

On the other hand, the biomass volumetric productivity value (using Equation (5)) (1.0 mg dm^−3^ h^−1^) was lower than the corresponding value obtained with sulfuric acid (3.0 mg dm^−3^ h^−1^) [29] and similar to that reached during the fermentation of phosphoric acid hydrolysates (0.9 mg dm^−3^ h^−1^) [28].

x = c + P_b_ t (5)

Yeasts usually tolerate only a narrow temperature range, and just two species, Kluyveromyces marxianus and *H. polymorpha*, have been reported to grow well above 40 °C. Nevertheless, the limited physiological data at elevated temperatures without corresponding metabolic analyses of the yeasts prevent a full understanding of this complex mechanism of thermotolerance. Whereas the maximum growth temperature was very similar in all strains investigated, the response of the metabolic network to elevated temperatures was not comparable between the different species. It was not possible to associate a specific behavior with the increase in temperature in the presence of *H. polymorpha* [51].

#### 3.2.2. Substrate Uptake

**Figure 3:**
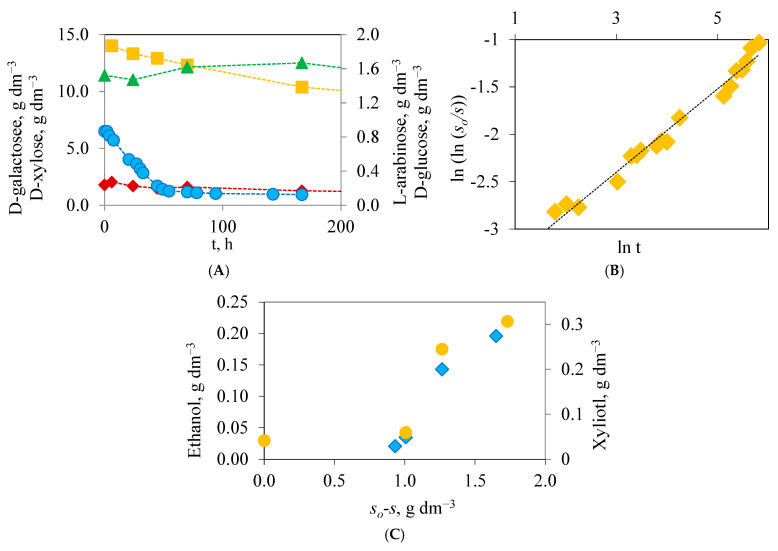
(**A**) Variation in D-xylose (■), D-glucose (●), D-galactose (♦), and L-arabinose (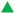) concentrations during the fermentation process carried out with hydrolysates of nitric acid. (**B**) Application of the linearization of Equation (6) to the fermentation process performed with hydrolysates of nitric acid. (**C**) Fit of the concentrations of ethanol (♦) and xylitol (●) versus sugar uptake (so-s) in the fermentation processes using hydrolysates of nitric acid.

The determination of sugar concentrations at the beginning of the fermentation process showed a higher availability of D-xylose (14.03 g dm^−3^) than that of D-glucose (0.82 g dm^−3^) and other sugars such as D-galactose (1.79 g dm^−3^) and L-arabinose (1.52 g dm^−3^) (Figure 3A). When the hydrolysate has been obtained with nitric acid, a sequential consumption of sugars is observed, what was also detected in previous studies [28,29] (Figure S5). To the nitric acid hydrolysate, it appears that *H. polymorpha* consumes D-glucose rapidly in the first stage of the culture, unlike the other sugars (Figure 3A).

The consumption of substrate was analyzed by determining the overall yields in biomass, $Y_{x/s}^{O}$, and the specific rate of substrate uptake, $q_{s}^{D}$ (Table 1).

The overall biomass yield throughout the experiment was calculated as the slope of the representation of the values (*x-x_o_*) at different times (*s_o_-s*). The biomass yield reached in the fermentation of nitric acid hydrolysate was 0.147 g g^−1^, which was a higher biomass yield than that achieved in the fermentation of sulfuric acid hydrolysate (0.17 g g^−1^) [29]. To determine the specific rate of substrate uptake the following expression was tested:

(6) $$S=S_{o}\alpha^{{-t}^{\beta }}$$

which fulfills the initial condition that *t* = 0 → *s* = *s_o_*, where *α* and *β* are empirical constants. The values of parameters *α* and *β* in Equation (6) can be obtained by linearization and making least-squares adjustments. By this, the derivative d(*s_o_-s*)/d*t* can be obtained analytically from Equation (6). Then the specific substrate uptake rate can be defined as a function of time [29]. Using this differential method, $q_{s}^{D}$ was established for each experiment (Figure 3B). In the case of nitric acid hydrolysate, the substrate took longer to consume ($q_{s}^{D}$ = 0.025 g g^−1^ h^−1^) than when other acids were used (Table 1 and Table S2).

For D-xylose consumption by yeast, the consumption was more moderate than for sulfuric acid hydrolysate. In another work, D-xylose consumption by the yeast strains took place once D-glucose was exhausted, leading to a long fermentation process and a slow and incomplete conversion of the sugars released from the lignocellulose hydrolysates [18]. In the first phase of culture, the increase in the consumption of sugars was more noticeable in the nitric acid hydrolysate than in the case of the other two acids (Figure 3B). Some authors have found the ability to assimilate nitrates by *H. polymorpha* [52,53], which may be related to the good fermentation results obtained with nitric acid. According to other researchers, the deficiency of D-xylose-specific transport proteins or the inhibition of D-xylose transporters by other sugars, especially by D-glucose, impedes the effective utilization of lignocellulose substrates [54]. This could justify the lower assimilation of D-xylose observed compared to the assimilation of D-glucose. Transport proteins were engineered to be less sensitive to D-glucose and more specific for D-xylose, with the aim of fostering the simultaneous uptake of sugars from lignocellulose substrates [18].

A simultaneous consumption of acetic acid by *H. polymorpha* to D-glucose and D-xylose uptake was detected, similar to that observed in another work [28]. Therefore, under the assumption that the substrate in the culture medium was made up of sugars and acetic acid, a new instantaneous biomass yield could be described, Yxs+AcO (Equation (7)), assuming also that both substrates had the same biomass yield:

(7) Yxs+Ac=dxdso−s+ACo−Ac

Equation (8) can be used to determine $q_{s+Ac}^{D}$, the specific substrate uptake rate including the acetic acid as substrate:

(8) $$\left(s-Ac\right)=\;\left(s_{o}+{Ac}_{o}\right)A^{{-t}^{B}}$$

When acetic acid was included as a substrate (Yxs+AcO), the biomass yield was 0.11 g g^−1^ for nitric acid hydrolysate and 0.54 g g^−1^ for the sulphuric acid hydrolysate. These values were higher than when only sugars as substrate were considered. The higher biomass yield when acetic acid acts as substrate (Yxs+AcO), compared to biomass yield in the case of sulphuric acid, could be justified by the simultaneous hexose utilization and flocculating behavior, providing reasons for the minor inhibition of sugar consumption observed in the presence of acetic acid. A more durable ability to resist the adverse inhibitory impact of acetic acid as exposure levels increase could explain the slightly faster sugar consumption rate shown when the acetic acid concentration is higher [55]. The specific substrate uptake rate including the acetic acid as substrate was superior in the case of nitric acid ($q_{s+Ac}^{D})$ = 0.048 g g^−1^ h^−1^) (Table 1) in comparison with values reached with sulfuric and phosphoric acid (Table S2).

Although *H. polymorpha* can grow and produce xylitol and ethanol even in the presence of inhibitors, in assays in organic clarified hemicellulose hydrolysates, the presence of higher amounts of inhibitors, including 12.24 g dm^−3^ acetic acid and 4.17 g dm^−3^ total phenolic compounds, affected the yeast performance greatly. These toxicity levels provoked an increase in the lag phase and reduced the D-xylose consumption rate. The clarification process eliminated impurities from the hydrolysate but provoked an increase in the concentration of inhibitors due to acidity and temperature conditions. During the fermentation with *H. polymorpha* with a synthetic medium, with D-glucose being the source of carbon, a biomass yield of 0.13 g g^−1^ and an ethanol yield of 0.42 g g^−1^ were reached, while with D-xylose as the carbon source, the results were 0.26 g g^−1^ and 0.12 g g^−1^ [49]. In the case of the hydrolysate with nitric acid, a biomass yield of 0.147 g g^−1^ and an ethanol yield of 0.25 g g^−1^ were reached (Table 1).

Comparing the fermentation process of the hydrolysate with nitric acid versus other acids, the higher growth of the microorganism could be attributed to a lower inhibitory effect, while the slower sugar consumption could be attributed to the possible utilization of acetic acid as a substrate by *H. polymorpha*.

#### 3.2.3. Bioproduct Formation

The formation of main bioproducts, ethanol and xylitol, were studied using the parameters ethanol ($Y_{E/s}^{0}$) and xylitol ($Y_{Xy/s}^{o}$) yields, and specific rates of ethanol ($q_{E}^{D}$) and xylitol ($q_{Xy}^{D}$) production (Table 1).

The instantaneous yield in xylitol, *Y_Xy/s_*, was calculated according to Equation (9):

(9) $$Y_{Xy/s}=\frac{d(Xy)}{d(S_{o}-S)}$$

considering that t = 0 → *Xy* = 0. If it remains constant throughout the experiment, it will represent an overall yield, which will be indicated by $Y_{Xy/s}^{o}$ (Equation (9)).

In the case of the ethanol, an instantaneous yield can be defined, *Y_E/s_*. If this yield remains constant during the culture, a representation of *E-E_o_*, or E (as in experiments *E_o_* = 0) vs. (*s_o_-s*) should lead to a straight line whose slope will correspond to the ethanol overall yield (YEso). Figure 3C shows the representations of the values of xylitol and ethanol concentrations versus sugar uptake.

The obtained values of ethanol ($Y_{E/s}^{0}$) and xylitol ($Y_{Xy/s}^{o}$) formation yields, as well as the specific xylitol ($q_{Xy}^{D}$) and ethanol ($q_{E}^{D}$) rates production, were higher for nitric acid than for sulfuric and phosphoric acid (Table S3), probably due to the very low amounts of phenolic compounds measured (Table S1), acting as inhibitors in the fermentation process with *H. polymorpha*. Working in the presence of *Dekkera bruxellensis* it has been observed that nitrate utilization affects carbon metabolism, and the yields of the products, and that acetic acid became the main product of D-glucose metabolism under aerobic conditions, instead of ethanol.

In addition, it was demonstrated that under anaerobic conditions, nitrate assimilation abolishes the “custers effect” (the inhibition of ethanolic fermentation in anaerobic conditions), improving its fermentative metabolism. This could be a new strategy to maintain growth and ethanol production for the use of this yeast in industrial processes [56]. According to this, the presence of nitrates could be related to the higher growth of the microorganism and the higher yield in bioproduct formation (Table 1). The best results obtained for nitric acid hydrolysate could be attributed to this ability of nitrate utilization by yeast, affecting carbon metabolism and the yields of the fermentation products. Some authors have indicated that the production of alcohols can provoke toxicity to the cell, which could limit productivity to industrial level [57]. Therefore, besides engineering pathways to propel the performance in carbon utilization and conversion, strategies to rise stress tolerance should be considered. The upper concentrations of acetic acid in the sulfuric acid fermentation can reduce significantly ethanol yield, due to unspecified toxicity from the effect of extracellular anions and inhibition of certain glycolytic enzymes caused by intracellular acidification and acetate accumulation [55]. This is in line with the results shown in Table S3.

The non-conventional yeast species exhibit atypical tolerance to stress in the bioethanol fermentation [58]. *H. polymorpha* grows and ferments at high temperatures; however, it produces reduced amounts of ethanol from D-xylose [59]. The non-conventional thermotolerant yeast *H. polymorpha* is able to naturally ferment D-xylose to ethanol at elevated temperatures (45 °C). The study of the molecular mechanisms involved in this metabolism is a hopeful route to increase the conversion of D-xylose to ethanol [60]. Other *H. polymorpha* strains ferment D-glucose, cellobiose, and D-xylose to ethanol at elevated temperatures (45–50 °C), with a relatively lower ethanol yield from D-xylose than from D-glucose and cellobiose. An increase in ethanol yield from D-xylose was obtained after targeted metabolic changes; even so, the final ethanol concentration achieved was still too low for an economic recovery by distillation [61]. The thermotolerant yeast *H. polymorpha*, in contrast to *S. cerevisiae*, can metabolize and ferment not only D-glucose but D-xylose, too. However, in non-conventional yeasts, the regulation of D-glucose and D-xylose metabolism remains poorly known [62].

Considering that higher temperatures promote the inhibitory effect of ethanol, placing the focus on the effect caused by using the three different acids, without inhibition in the production of ethanol, the thermotolerance characteristic of *H. polymorpha* has not been used. No relevant results were found by increasing the temperature when the hydrolysate was H_2_SO_4_ [29]. The study of the isolation and selection of microorganisms capable of adapting better to these severe conditions must be continued [63].

### 3.3. Solid Fraction

The solid fractions in which a lower hemicellulosic fraction and an upper cellulosic fraction remained after acid pretreatment were characterized. These solid fractions corresponded to the hydrolysates of nitric acid 1 mol dm^−3^, sulfuric acid 0.5 mol dm^−3^, and phosphoric acid 2.67 mol dm^−3^.

#### 3.3.1. Fourier Transform Infrared (FTIR)

**Figure 4:**
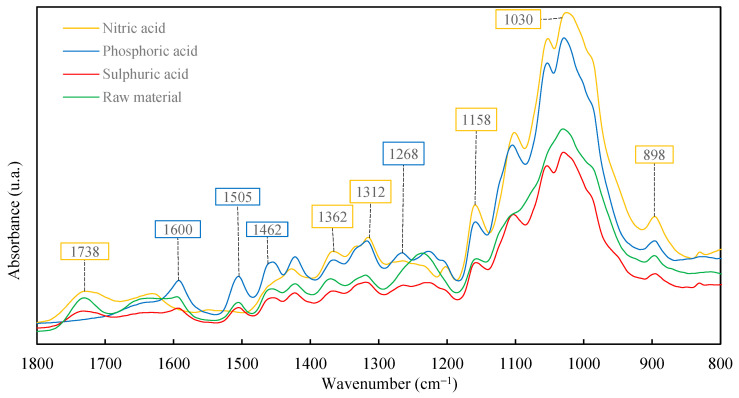
FTIR of raw material and solids treated with the three acids.

This analysis was conducted to research the changes in organic functional groups in the biomass samples treated with different acids. The degradation provoked reduction in the relative intensities of lignin peaks at 1505, 1462, 1244, and 1125 cm^−1^. The changes in the intensities of carbohydrate bands at 1375, 1158, and 898 cm^−1^ were less marked (Figure 4). The absorbance in the region from 1200 to 900 cm^−1^, which is the polysaccharide region [64,65], was sharply decreased in lignin [66].

Distinct vibration bands were connected to cellulose, viz., the asymmetric C–O–C bridge stretching vibration at 1.157 cm^−1^ and the C–H bending vibration at 1362 cm^−1^. The intensities of these peaks were high and sharp. A cellulose vibration (CH_2_ wagging vibration) at 1312 cm^−1^ was also visible, oriented vertically to the cellulose chain [67]. For the lignin, the characteristic band signals (1505 and 1600 cm^−1^) diminished for the residue treated with nitric acid. Degradation gave rise to a significant decrease in the intensities of polysaccharide bands at 1738 cm^−1^, 1375 cm^−1^, 1158 cm^−1^, and 898 cm^−1^. This decease occurred with a rise in the relative intensities of absorption bands due to lignin at 1596 cm^−1^, 1505 cm^−1^, 1462 cm^−1^, 1268 cm^−1^, 1245 cm^−1^, and 1125 cm^−1^ [68].

The FTIR analysis showed the predominance of the cellulosic fraction for the residue treated with nitric acid.

#### 3.3.2. Scanning Electronic Microscopy (SEM)

**Figure 5:**
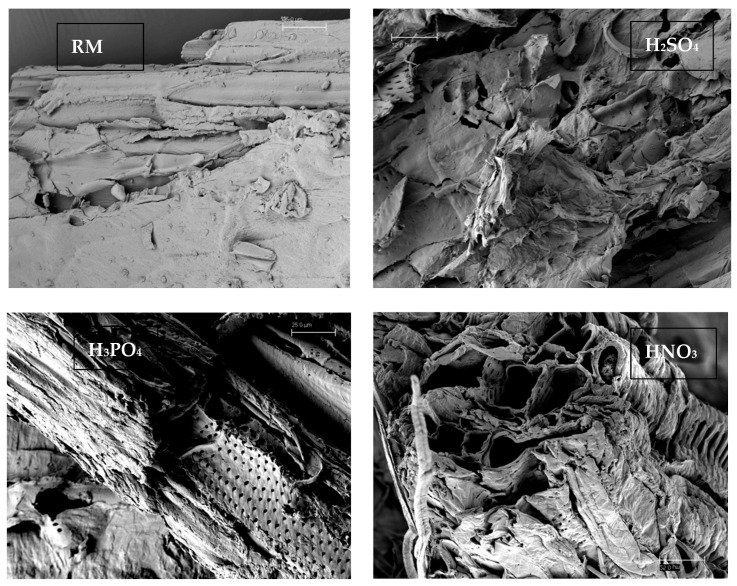
SEM of raw material and solids treated with the three acids.

SEM is a non-destructive material characterization technique that evaluates the fundamental microstructural characteristics of materials, such as surface topography, grain size, and local chemistry, providing an overview of the consequences of applied treatments [69] (Figure 5).

The SEM image showed, for the case of the residue pretreated with nitric acid, a greater disruption than the one treated with the other acids caused by the high elimination rate of lignin and hemicellulose. Figure 5 shows the effect of the acid treatment in the lignocellulosic solids studied. In the case of the solid pretreated with nitric acid, the aspect presented could be attributed to the loss of lignin.

#### 3.3.3. Elemental Analysis and Higher Heating Values

Although there was an increase of 13.18% in the HHV of the solid pretreated with nitric acid with respect to that of the original biomass, the value of this parameter was lower than that achieved when the solid was treated with other acids (Table 2), probably due to the lower % of C in the case of the solid treated with nitric acid.

### 3.4. Enzymatic Hydrolysis

In order to increase this concentration in D-glucose, corresponding to the non-hydrolyzed cellulosic fraction, a subsequent enzymatic hydrolysis step was applied to the acid pretreated solids. The acid treatment application meant the elimination of most of the lignin and 100% of the hemicellulose, allowing high accessibility to the cellulosic fraction sugars (89.8%), resulting in a high yield in the production of sugars, according to results reached in other research [70]. The values of cellulose % of the original residue and the sunflower stalk residue treated with water were 32% and 53.6%, respectively. In the case of pretreatments with phosphoric acid [28] and sulfuric acid [29], the percentage of cellulose obtained was 63.3% and 59.4%, respectively.

The solid yield (g per 100 g raw material) in the case of enzymatic hydrolysis on the residue treated with nitric acid was 41.1%, recovering more solid when the pretreatments were performed with phosphoric (69.12%) and sulfuric acid (77.6%), recovering 87.03% after enzymatic hydrolysis of the untreated residue (Table 3).

**Figure 6:**
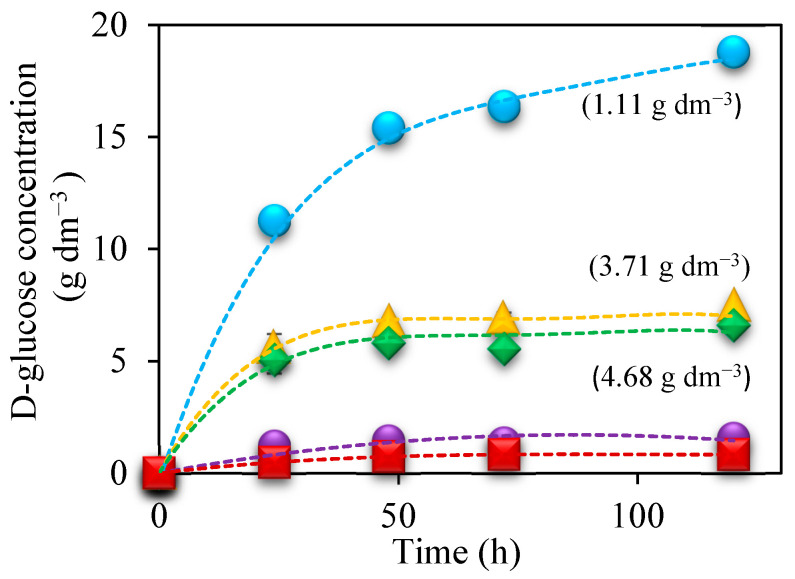
Variation in D-glucose concentration during the enzymatic hydrolysis for the residues of nitric acid (●), phosphoric acid (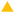), sulfuric acid (♦), raw material (●), and raw material without enzyme (■). In brackets: xylose concentration during the enzymatic hydrolysis for each acid.

Figure 6 shows how it is possible to recover more D-glucose and less D-xylose after enzymatic treatment in the case of the solid previously treated with nitric acid compared with when the other two acids are used, which means, for this nitric acid pretreated solid, increasing the degree of utilization for further fermentation instead of using it for energy recovery. For this type of energy use, the solids treated with H_2_SO_4_ or HNO_3_ would be more suitable. A subsequent enzymatic hydrolysis carried out on the residue pretreated with nitric acid would show a greater generation of cellulosic glucose, and, therefore, greater utilization of this fraction, which would improve the results achieved in the fermentation of the acid hydrolysate.

In the case of other biomasses such as artichoke, after the hydrolysis and fermentation of 5% (*w*/*v*) HNO_3_-pretreated Jerusalem artichoke stalks, an upper concentration of glucose and ethanol led to 38.5 g dm^−3^ of glucose after saccharification. This value agrees with the 89% theoretical enzymatic hydrolysis yield, and 9.5 g dm^−3^ of ethanol [71], versus 18.4 g dm^−3^ of ethanol reached after enzymatic hydrolysis of the solid fraction of sunflower stalks pretreated with nitric acid.

The use of industrial-scale nitric acid pretreatment can have adverse consequences for the environment, such as acidification of soil and water by acids and nitrates; therefore, the design of the processes must take into account the implementation of the prevention and environmental protection measures required.

## 4. Conclusions

In this work, we studied acid hydrolysis with nitric acid on sunflower stalk residues followed by fermentation of the hydrolysate obtained. Although sunflower stalk residue does not generate as many sugars as other biomasses, such as corncob, a valorization of this biomass is possible when using the appropriate acid. The fermentation process developed with *H. polymorpha* exhibited promising results by virtue of its specific properties, especially its resistance to inhibition and its ability to co-ferment both D-glucose and D-xylose, which seems to be particularly enhanced in nitric acid hydrolysis, eliminating the need to resort to the thermotolerance of the microorganism. The nitric acid hydrolysis of sunflower stalks showed the highest rate of hydrolysis (21.12 g dm^−3^ h^−1^) and the lowest concentrations of inhibitors in comparison with other acid treatments. During fermentation in the presence of *H. polymorpha*, the nitric acid hydrolysate reached the higher maximum specific growth rate, *μm* = 0.12 h^−1^, and the lowest consumption of substrate rate ($q_{S}^{D}$ = 0.025 g g^−1^ h^−1^) compared to fermentation processes of hydrolysates with sulfuric and phosphoric acids. In relation to alcohols, likewise, the best $Y_{E/S}^{0}$, $Y_{Xy/S}^{0}$ values were reached in the case of the nitric acid hydrolysate, and a higher D-glucose consumption than D-xylose was observed. Relevant perspectives were presented for the use of lignocellulose residues to obtain bioalcohols using non-conventional yeasts such as *H. polymorpha* in the fermentation processes of hydrolysates, deepening our knowledge of the influencing factors implicated in the metabolic pathways.

A characterization of the solid fraction obtained after hydrolyzing with acids (sulfuric, phosphoric, and nitric) was enacted in order to try to justify the different hydrolysis and fermentation results found, depending on the acid used. The solid fraction resulting from nitric acid hydrolysis possessed a higher cellulose content than that obtained when the pretreatment was developed with other acids. This is why the pretreatment with nitric acid prior to enzymatic hydrolysis increased the availability of cellobiotic glucose to a greater extent than in the case of the other acids.

Hydrolysates of sunflower stalk residues pretreated with nitric acid offered better results during the fermentation process in the presence of *H. polymorpha* than those obtained with sulfuric acid or phosphoric acid. For an energetic utilization of the solid fraction, the solids treated with sulfuric and phosphoric acids gave higher HHVs than those treated with nitric acid. However, the posterior enzymatic hydrolysis of the solids pretreated with nitric acid generated a higher amount of D-glucose than that achieved after enzymatic hydrolysis with the other acids. This indicates that it is possible to increase the utilization of sunflower stalk residues by pretreatment of the biomass with nitric acid, followed by additional enzymatic hydrolysis for a greater utilization of the cellulosic fraction, leading to a better performance during fermentation developed by *H. polymorpha*, than when other acids are used.

## Figures and Tables

**Table 1:** Kinetic parameters during the fermentation of nitric acid hydrolysates.

Overall Biomass Yields and Specific Rates of Substrate Uptake		Specific Rates and Overall Yields and of Bioproducts Formation	
Yxso, g g^−1^	0.147	$q_{xy}^{D}$, g g^−1^	0.0091 (50 h)
$q_{s}^{D}$, g g^−1^ h^−1^	0.025 (50 h)	$q_{E}^{D}$, g g^−1^ h^−1^	0.055 (30 h)
Yxs+AcO, g g^−1^	0.11	$Y_{Xy/s}^{0}$, g g^−1^	0.14
$q_{s+Ac}^{D}$, g g^−1^ h^−1^	0.048 (50 h)	$Y_{E/s}^{O}$, g g^−1^ h^−1^	0.25

**Table 2:** Elemental analysis and HHVs.

Samples	Nitrogen, %	Carbon, %	Hydrogen, %	Sulfur, %	HHV, MJ kg^−1^
Raw material	0.239 ± 0.002	44.781 ± 0.151	5.911 ± 0.001	1.976 ± 0.050	15.413 ± 0.05
H_2_SO_4_	0.204 ± 0.000	45.796 ± 0.493	5.836 ± 0.005	3.180 ± 0.023	19.124 ± 0.094
H_3_PO_4_	0.182 ± 0.000	46.939 ± 0.000	5.858 ± 0.001	2.589 ± 0.046	19.161 ± 0.052
HNO_3_	0.129 ± 0.005	42.535 ± 0.020	5.879 ± 0.004	2.251 ± 0.090	17.444 ± 0.154

**Table 3:** Enzymatic hydrolysis yield and % solid recovered percentage.

Samples	24 h	48 h	72 h	120 h	% Solid Recovery
RMWE ^1^	-	-	-	1.407 ± 0.127	82.688 ± 0.530
RM ^2^	0.118 ± 0.041	0.211 ± 0.007	0.227 ± 0.029	1.976 ± 0.050	87.0367 ± 0.736
H_2_SO_4_	4.601 ± ND	7.026 ± ND	7.016 ± ND	19.774 ± 0.042	77.650 ± 1.218
H_3_PO_4_	9.958 ± ND	21.965 ± ND	30.995 ± ND	21.367 ± 0.648	69.126 ± 1.156
HNO_3_	12.067 ± ND	16.114 ± ND	16.900 ± ND	37.446 ± 1.694	41.095 ± 0.219

^1^ Raw material without enzyme. ^2^ Raw material with enzyme.

## Data Availability

The original contributions presented in this study are included in the article/Supplementary Materials. Further inquiries can be directed to the corresponding author.

## Supplementary Materials

The following supporting information can be downloaded at: https://www.mdpi.com/article/10.3390/polym16243548/s1, Figure S1: Variation in the concentrations of total reducing sugars (TRSs) (a) and D-glucose (b) produced throughout hydrolysis process with phosphoric acid 2.67 mol dm^−3^ (), nitric acid 1.0 mol dm^−3^ (♦), sulfuric acid 0.5 mol dm^−3^ (), and sulfuric acid 2.5 mol dm^−3^ (); Figure S2: Total reducing sugars (TRSs) (a) and D-glucose (b) yields (referred to initial dry material) versus initial concentration of phosphoric acid (), nitric acid (♦), and sulfuric acid () used in the hydrolysis process; Figure S3: Variation in the hemicellulose (), cellulose (), and lignin () percentages with the initial concentration of sulfuric acid (a), phosphoric acid (b), and nitric acid (c) used in the hydrolysis process; Figure S4: Influence of phosphoric acid (), nitric acid (♦), and sulfuric acid () concentrations on hemicellulose fractional conversion (a) and cellulose fractional conversion (b); Figure S5: Variation in sugar concentrations (D-xylose, D-glucose, D-galactose, L-arabinose) during the fermentation process carried out with hydrolysates of sulfuric acid (), phosphoric acid (), and nitric acid (♦); Figure S6: Biomass formation in the fermentation processes of hydrolysates of sulfuric acid (), phosphoric acid (), and nitric acid (♦); Table S1: Phenolic compounds (PCs) and acetic acid concentrations; Table S2: Overall biomass yields and specific rates of substrate uptake; Table S3: Kinetic parameters in the processes of bioproducts formation.

## Nomenclature

α	Adjustment parameter in Equation (6)
β	Adjustment parameter in Equation (6)
$C_{Ao}$	Initial acid concentration (g dm^−3^)
E	Ethanol concentration (g dm^−3^)
E_T_	Maximum theoretical concentration attainable of ethanol (g dm^−3^)
*f*	Residual concentration of D-fructose (g dm^−3^)
*g*	Residual concentration of D-glucose (g dm^−3^)
*P_b_*	Volumetric biomass productivity (mg dm^−3^ h^−1^)
$q_{E}^{D}$	Specific rate of ethanol production (mg g^−1^ h^−1^)
$q_{s}^{D}$	Specific rate of sugar consumption (mg g^−1^ h^−1^)
$q_{s+Ac}^{D}$	Specific rate of sugar and acetic acid consumption (mg g^−1^ h^−1^)
$q_{Xy}^{D}$	Specific rate of xylitol production (mg g^−1^ h^−1^)
*r_o_*	Initial hydrolysis rate (g dm^−3^ h^−1^)
*s*	Residual concentration of total sugars (g dm^−3^)
*s_o_*	Initial concentration of total sugars (g dm^−3^)
*s_m_*	Maximum total sugars concentration (g dm^−3^)
*t*	Time (h)
*TRS*	Total reducing sugars (g dm^−3^)
*x*	Biomass concentration (g dm^−3^)
*x_o_*	Initial biomass concentration (g dm^−3^)
*X*	Fractional conversion
*Xy*	Xylitol concentration (g dm^−3^)
$Y_{E/s}^{O}$	Overall ethanol yield (g g^−1^)
*Y _E/s_*	Instantaneous ethanol yield (g g^−1^)
*Y_G_*	D-glucose yield in the hydrolysis (g g^−1^)
$Y_{x/s}^{O}$	Overall biomass yield (g g^−1^)
*Y_T_*	Total sugars yield in the hydrolysis (g g^−1^)
$Y_{xy/s}^{O}$	Overall xylitol yield (g g^−1^)
*Y _xy/xyl_*	Instantaneous xylitol yield (g g^−1^)
*µ_m_*	Maximum specific growth rate (h^−1^)
