# Supplementary material for: Harnessing of Sunflower Stalks by Hydrolysis and Fermentation with Hansenula polymorpha to Produce Biofuels
Source: Polymers (Basel). 2024 Dec 19;16(24):3548. doi: 10.3390/polym16243548 (PMC11678814; doi:10.3390/polym16243548)
Supplement: Supplementary file 1 [file polymers-16-03548-s001.zip › polymers-3261321-supplementary.pdf]

Figures. Supplementary Information

Figure S1

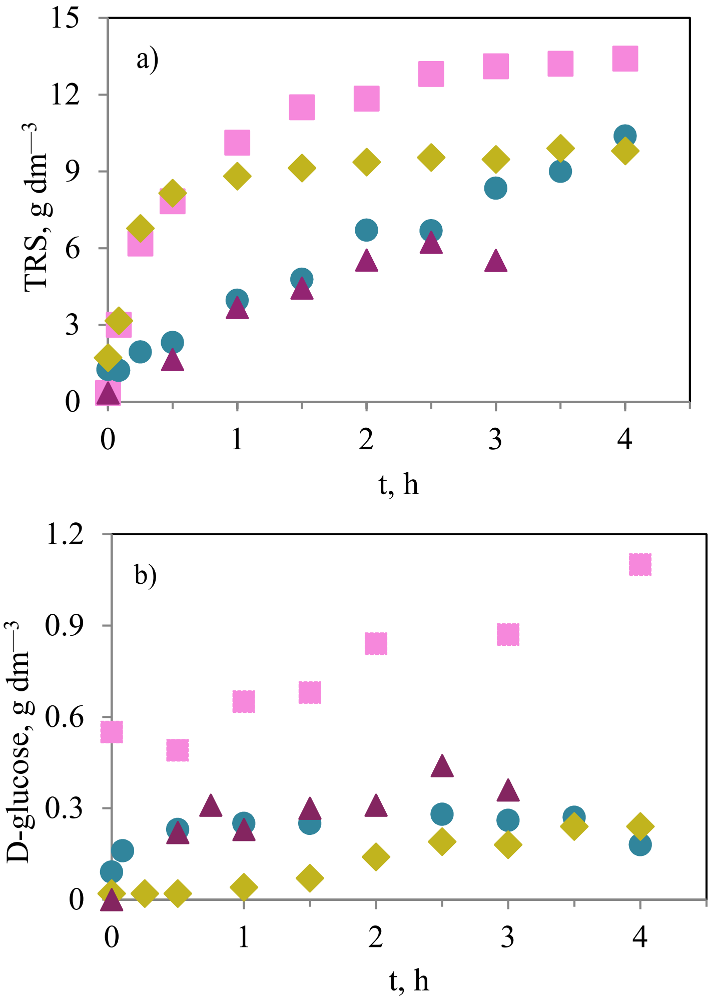

Figure S1. Variation in the concentrations of total reducing sugars (TRS) (a) and D-glucose (b) produced throughout hydrolysis process with phosphoric acid 2.67 mol dm^{-3} (●), nitric acid 1.0 mol dm^{-3} (◆), sulphuric acid 0.5 mol dm^{-3} (▲) and sulphuric acid 2.5 mol dm^{-3} (■).

Figure S2

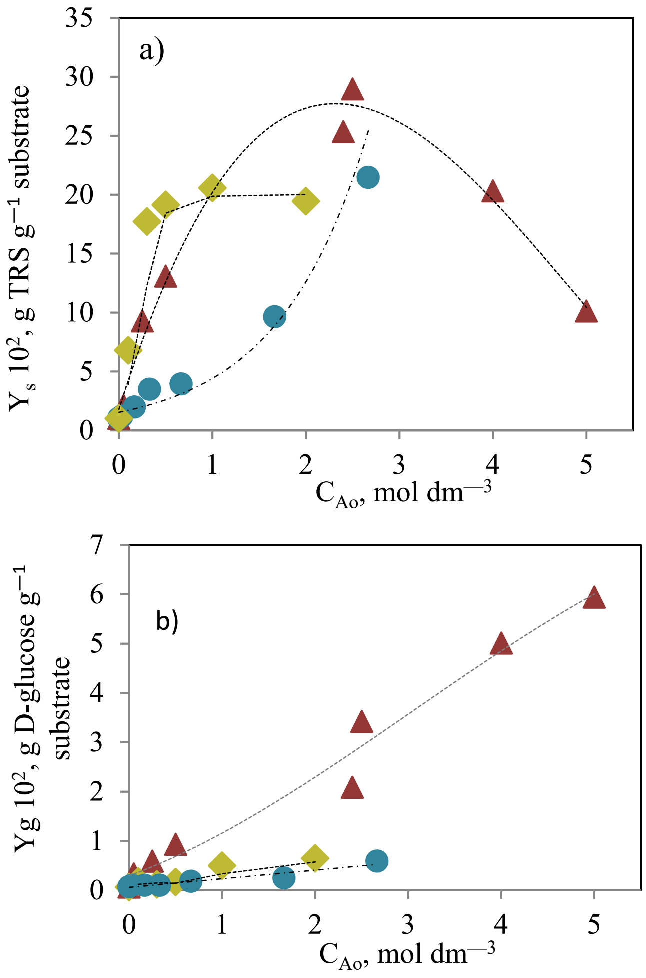

Figure S2. Total reducing sugars (TRS) (a) and D-glucose (b) yields (referred to initial dry material) *versus* initial concentration of phosphoric acid (●), nitric acid (◆) and sulphuric acid (▲) used in the hydrolysis process.

Figure S3

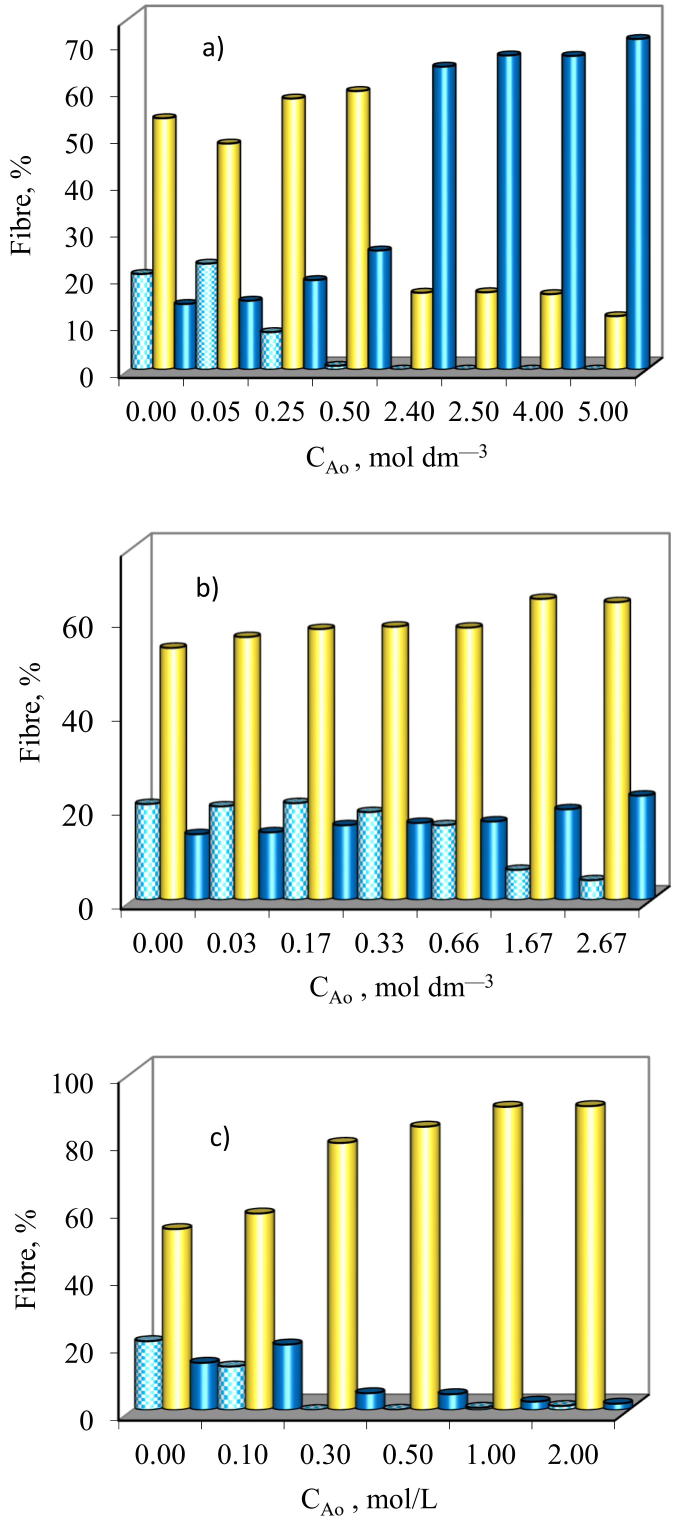

Figure S3. Variation of the hemicellulose ( ), cellulose ( ) and lignin ( ) percentages with the initial concentration of sulphuric acid (a), phosphoric acid (b) and nitric acid (c) used in the hydrolysis process.

Figure S4

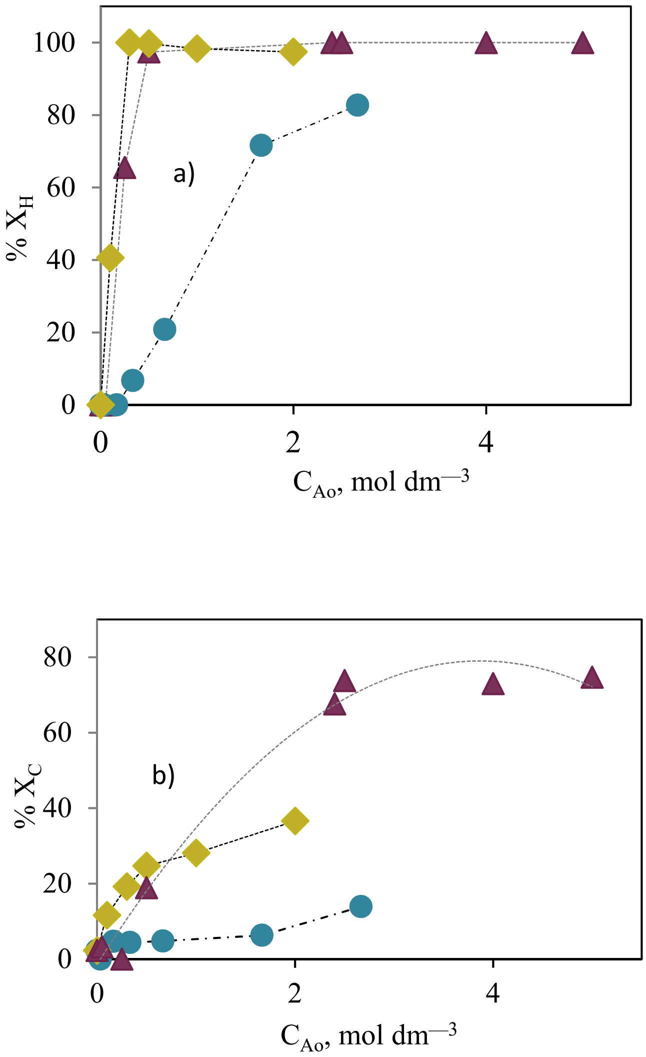

Figure S4. Influence of phosphoric acid (●), nitric acid (◆) and sulphuric acid (▲) concentrations on hemicellulose fractional conversion a) and cellulose fractional conversion b).

Figure S5

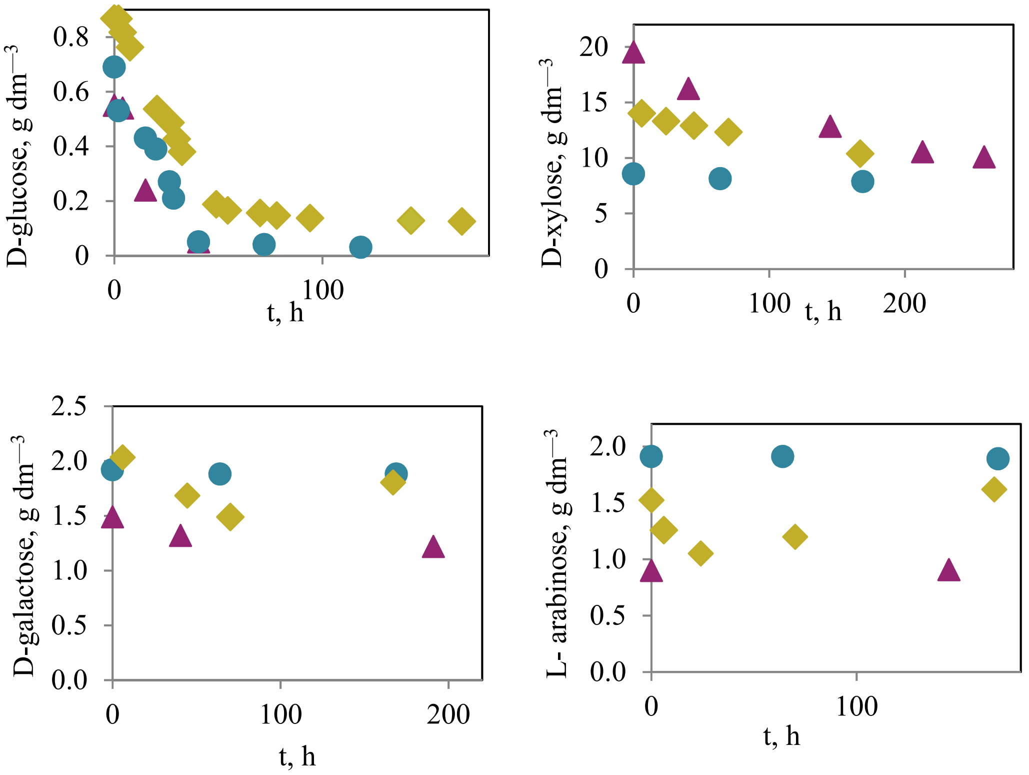

Figure S5 Variation of sugar concentrations (D-xylose, D-glucose, D-galactose, L-arabinose) during the fermentation process carried out with hydrolysates of sulphuric acid (▲), phosphoric acid (●) and nitric acid (◆).

Figure S6

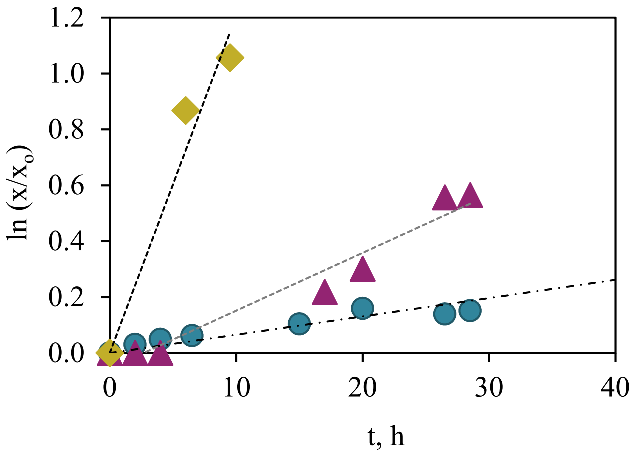

Figure S6 Biomass formation in the fermentation processes of hydrolysates of sulphuric acid (▲), phosphoric acid (●) and nitric acid (◆).

Tables

Table S1. Phenolic compounds (PC) and acetic acid concentrations

$mg\ dm^{-3}$	Nitric acid
Hydroxytyrosol	0.013
Vanillin	ND
Veratric acid	ND
Syringic acid	0.009
Vanillic acid	ND
Protocatechuic acid	0.001
Caffeic acid	ND
Ferulic acid	ND
t-Cinamic acid	0.004
p-Bromophenol	0.003
Total PC	0.030
Acetic Acid	2.1

ND: Not Detected

Table S2. Overall biomass yields and specific rates of substrate uptake

	**Sulphuric acid**	**Phosphoric acid**	**Nitric acid**
$Y^{O}_{x/s}$, $g\ g^{-1}$	0.17	0.103	0.147
q^{D}_{s}, $g\ g^{-1}\ h^{-1}$	0.067(50 h)	0.044(50 h)	0.025(50 h)
$Y^{O}_{x/s+Ac}$, $g\ g^{-1}$	0.540	0.080	0.110
q^{D}_{s+Ac}, $g\ g^{-1}\ h^{-1}$	0.0065 (595 h)	0.023 (310 h)	0.048 (50 h)

Table S3. Kinetic parameters in the processes of bioproducts formation

	**Sulphuric acid**	**Phosphoric acid**	**Nitric acid**
q^{D}_{xy}, $g\ g^{-1}\ h^{-1}$	0.003 (64 h)	0.00014 (30 h)	0.009 (50 h)
q^{D}_{E}, $g\ g^{-1}\ h^{-1}$	0.022 (30 h)	0.014(30 h)	0.055 (30 h)
$Y^{O}_{Xy/s}$, $g\ g^{-1}$	0.12	0.023	0.14
$Y^{O}_{E/s}$, $g\ g^{-1}$	0.071	0.14	0.25
